# Supplementary material for: Splice-Junction-Based Mapping of Alternative Isoforms in the Human Proteome
Source: Cell Rep. Author manuscript; Available in PMC 2020 Jan 15. (PMC6961840; doi:10.1016/j.celrep.2019.11.026)
Supplement: 3 [file NIHMS1546469-supplement-3.zip › DF2/PXD0010154/Testis-198-Q96PU5-SCSVTDAVAEQGHLPPPSVAYVHTTPGLPSGWEER.pdf]

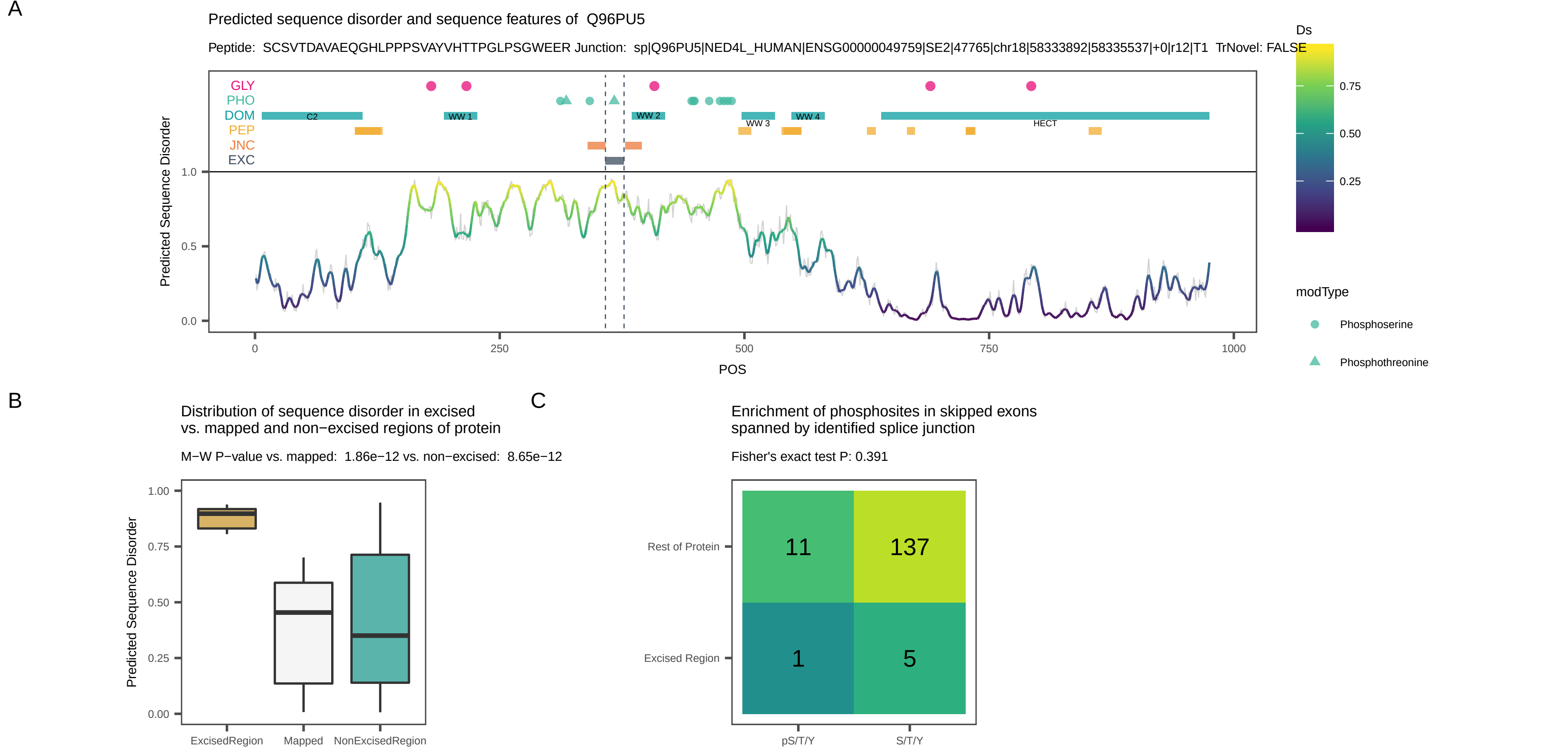
A
Predicted sequence disorder and sequence features of Q96PU5
Peptide: SCSVTDAVAEQGHLPPPSVAYVHTTPGLPSGWEER Junction: sp|Q96PU5|NED4L_HUMAN|ENSG00000049759|SE2|47765|chr18|58333892|58335537|+0|r12|T1 TrNovel: FALSE
GLY
PHO
DOM
PEP
JNC
EXC
C2
WW 1
WW 2
WW 3
WW 4
HECT
Predicted Sequence Disorder
1.0
0.5
0.0
0
250
500
750
1000
POS
Ds
0.75
0.50
0.25
modType
Phosphoserine
Phosphothreonine
B
Distribution of sequence disorder in excised
vs. mapped and non−excised regions of protein
M−W P−value vs. mapped: 1.86e−12 vs. non−excised: 8.65e−12
Predicted Sequence Disorder
1.00
0.75
0.50
0.25
0.00
ExcisedRegion
Mapped
NonExcisedRegion
C
Enrichment of phosphosites in skipped exons
spanned by identified splice junction
Fisher's exact test P: 0.391
Rest of Protein
Excised Region
11
137
1
5
pS/T/Y
S/T/Y
